# Learning curve of trans-sacral epiduroscopic laser decompression in herniated lumbar disc disease

**DOI:** 10.1186/s12893-020-00949-8

**Published:** 2021-01-18

**Authors:** Seong Son, Chan Jong Yoo, Byung Rhae Yoo, Woo Seok Kim, Tae Seok Jeong

**Affiliations:** Department of Neurosurgery, Gil Medical Center, Gachon University College of Medicine, #24, 74th Street, Namdongdaero Namdong-Gu, Incheon, 405-220 South Korea

**Keywords:** Learning curve, Disc, Lumbar spine, Trans-sacral epiduroscopic laser decompression, Minimally invasive surgery

## Abstract

**Background:**

Trans-sacral epiduroscopic laser decompression (SELD) using slender epiduroscope and a holmium YAG laser is one of the minimally invasive surgical options for lumbar disc herniation. However, the learning curve of SELD and the effect of surgical proficiency on clinical outcome have not yet been established. We investigated patients with lumbar disc herniation undergoing SELD to report the clinical outcome and learning curve.

**Methods:**

Retrospective analysis of clinical outcome and learning curve were performed at a single center from clinical data collected from November 2015 to November 2018. A total of 82 patients who underwent single-level SELD for lumbar disc herniation with a minimum follow-up of 6.0 months were enrolled. Based on the findings that the cut-off of familiarity was 20 cases according to the cumulative study of operation time, patients were allocated to two groups: early group (n = 20) and late group (n = 62). The surgical, clinical, and radiological outcomes were retrospectively evaluated between the two groups to analyze the learning curve of SELD.

**Results:**

According to linear and log regression analyses, the operation time was obtained by the formula: operation time = 58.825–(0.181 × [case number]) (p < 0.001). The mean operation time was significantly different between the two groups (mean 56.95 min; 95% confidence interval [CI], 49.12–64.78 in the early group versus mean 45.34 min; 95% CI, 42.45–48.22 in the late group; p = 0.008, non-parametric Mann–Whitney U test). Baseline characteristics, including demographic data, clinical factors, and findings of preoperative magnetic resonance imaging, did not differ between the two groups. Also, there was no significant difference in terms of surgical outcomes, including complication and failure rates, as well as clinical and radiological outcomes between the two groups.

**Conclusion:**

The learning curve of SELD was not as steep as that of other minimally invasive spinal surgery techniques, and the experience of surgery was not an influencing factor for outcome variation.

## Background

Lumbar epiduroscopy, also known as lumbar epidural endoscopy, is a minimally invasive percutaneous procedure to assess the epidural space thorough the sacral hiatus. Trans-sacral epiduroscopic decompression (SELD), involving the use of a small-caliber flexible epiduroscope and laser technology, was developed in early 2000s [1]. SELD has been clinically used in lumbosacral spine diseases for direct visual diagnosis and treatment of epidural pathology, including disc herniation, spinal stenosis, and epidural space adhesion [2–7].

Recently, based on the principle of lasers to condense the hydrated herniated disc, mild to moderate soft disc herniation is considered as the optimal indication of SELD [8, 9]. Accordingly, several previous studies have reported its various clinical outcome and safety in lumbar disc herniation [8–11]. In addition, SELD is considered to be an easier procedure than classical full endoscopic spinal surgery [8]. However, there is a definite threshold for a skillful surgical technique in terms of the approach via the sacral hiatus, safe entering into the ventral epidural space, reaching the target site by a flexible endoscope, and utilizing a very narrow and magnified endoscopic view. Accordingly, we assumed that the learning curve of a surgeon to SELD, that is whether the surgeon is a beginner or expert of SELD, might affect the clinical outcome.

However, there are no reports about the learning curve of SELD and the effect of surgical proficiency on clinical outcome. In this paper, we analyzed the learning curve of SELD and evaluated outcomes based on the degree of surgical skill in patients with lumbar disc herniation.

## Methods

### Indications and patient population

As described in author’s previous article [9], the indication for SELD was mild to moderate soft disc herniation with concordant low back pain and/or radicular leg pain despite conservative management (medication, physiotherapy, or nerve block) at least 2 weeks or concordant severe pain making daily life activities impossible. The contraindication included disc herniation with motor weakness, calcified disc herniation, inaccessible foraminal disc herniation, spinal stenosis or instability, infection, limited blood coagulation symptom, anomaly of sacral hiatus, or peridural cyst.

Between November 2015 and November 2018, among 116 patients who underwent SELD by one surgeon in a single institution, 82 patients were selected and data analysis was performed retrospectively. To minimize the bias of patient selection, study inclusion criteria were as follows: (1) single-level disc herniation, (2) no history of surgery in the lumbar spine, and (3) at least 6 months of follow-up with complete medical record.

All surgical procedures were performed by one surgeon via sacral hiatus under local anesthesia according to the previously reported methods [9].

### Learning curve evaluation based on the operation time

The authors assessed the learning curve of SELD by analyzing the operation time. The operation time was defined as the duration from the skin incision to skin closure. The authors recorded the operation time according to case number, and linear regression analyses was performed to reveal the linear correlation between operation time and case series number. Furthermore, cumulative analysis of operation time was performed in order to confirm a cut-off value of familiarity.

### Outcome evaluation

**Fig. 1:**
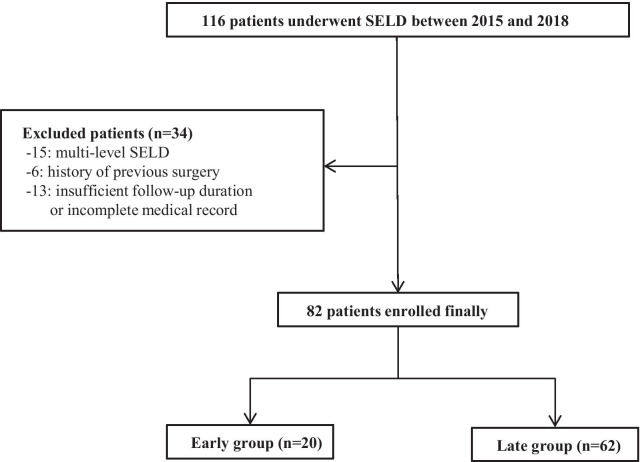
Final study cohort selection. SELD, trans-sacral epiduroscopic laser decompression

We performed retrospective analysis in terms of baseline characteristics, surgical outcome, clinical outcome, and radiological outcome as depicted in author’s previous article [9]. Based on the significant cut-off value of operation time according to the cumulative analysis of operation time, the final cohort was divided into two groups: the early group including the earlier 20 cases and the late group including the later 62 cases (Fig. 1).

**Fig. 2:**
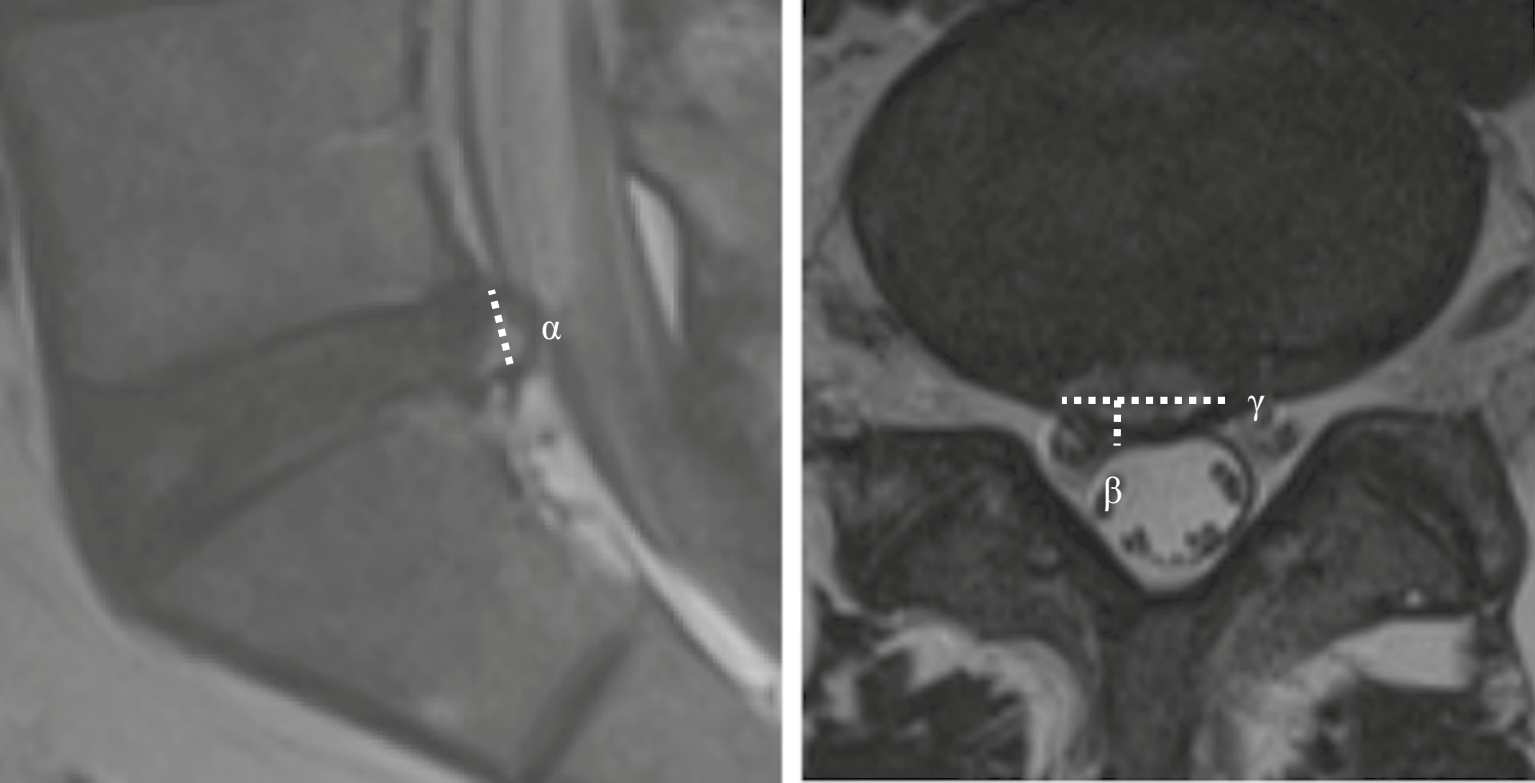
The protruded disc volume was determined as height (α) × depth (β) × transverse diameter (γ) of disc herniation × 1/2 (mm^3^) in preoperative magnetic resonance imaging

Demographic data, such as age, sex, body mass index, and clinical baseline characteristics, such as past medical history, preoperative duration of symptom, previous history of nerve block, trauma history, and symptom dominance (low back pain or radiating leg pain) were assessed between the two groups. In addition, preoperative magnetic resonance imaging (MRI) findings, such as degree of disc degeneration and degree of herniation, were evaluated between the two groups. The volume of disc herniation was determined as transverse diameter × depth × height of disc herniation × 1/2 (mm^3^) (Fig. 2).

Surgical outcomes, including complication rate and failure or recurrence rate, were evaluated between the two groups. The failure or recurrence rate was assessed using an additional procedure rate including revision surgery or nerve block during 6 months after surgery.

Clinical outcomes, including visual analogue scale (VAS) scores of low back pain and leg pain, and patient satisfaction using Odom’s criteria, were surveyed at each follow-up visits (1 week, 1 month, and 6 months after surgery) between the two groups.

**Fig. 3:**
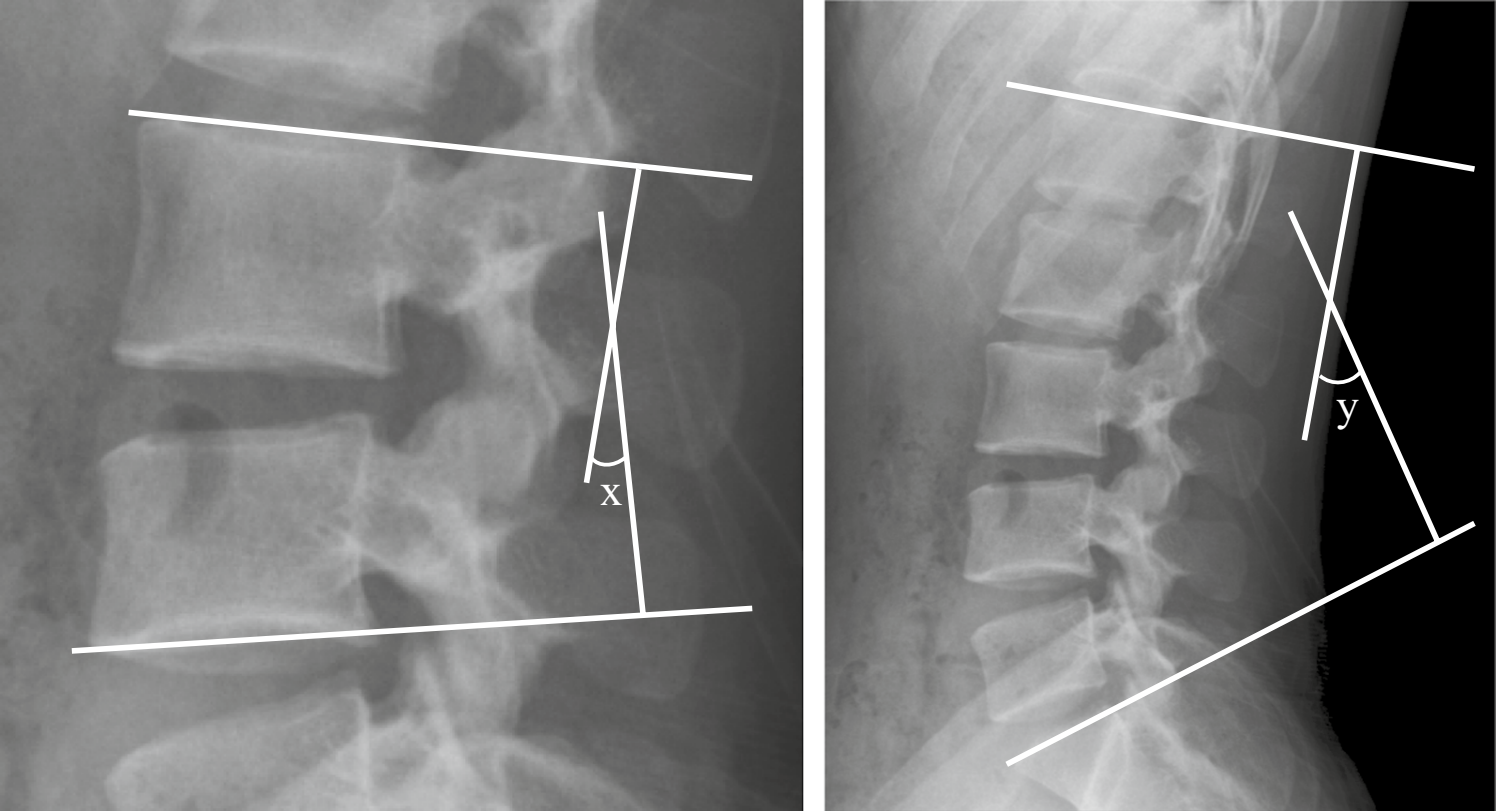
Lateral plain radiograph of the lumbar spine showing measured variables. Segmental angle (x) and total lumbar lordosis (y) were defined at the intersection of lines

Plain radiographs were performed preoperatively and at 6 months, and we analyzed the radiological change in lumbar alignment between the two groups. Segmental angle and range of motion at the surgery level as well as total lumbar lordosis were assessed using Cobb’s method (Fig. 3).

### Statistical analysis

Data management and statistical analysis were performed using SPSS version 23.0 (SPSS Inc., Chicago, IL, USA). We performed a cumulative study and linear regression analysis to analyze the learning curve based on operation time. In addition, Pearson’s chi square test, non-parametric Mann–Whitney U test, and independent t-test were used according to the characteristics of the factors to identify differences between the two groups. Results are expressed as means ± standard deviations or means with 95% confidence intervals (CIs), and statistical significance was considered for p values of < 0.05.

## Results

### Learning curve of SELD based on operation time

**Fig. 4:**
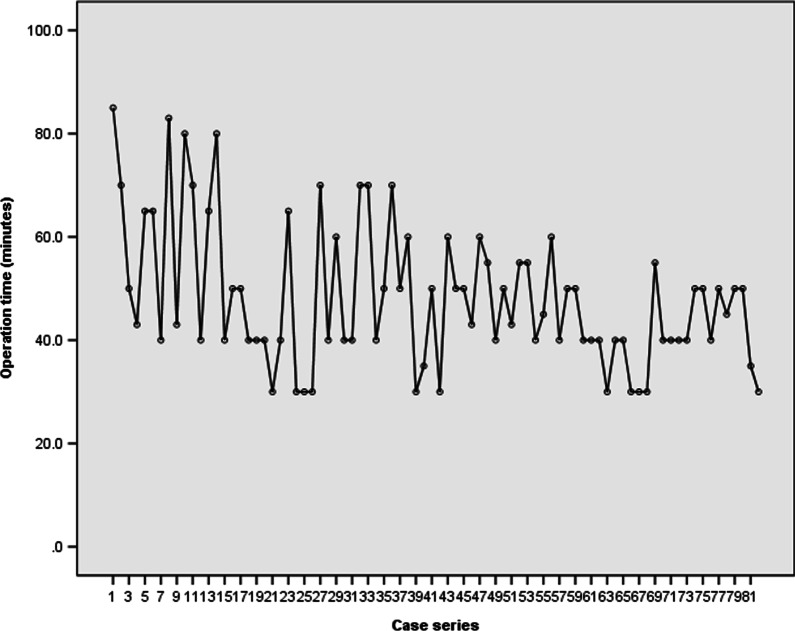
Operation time according to case series number

The mean operation time was 56.07 (95% CI, 48.18–63.97) minutes among all patients. There was a trend of decreasing operation time with an accumulation of case series or surgical experience of the surgeon (Fig. 4).

**Fig. 5:**
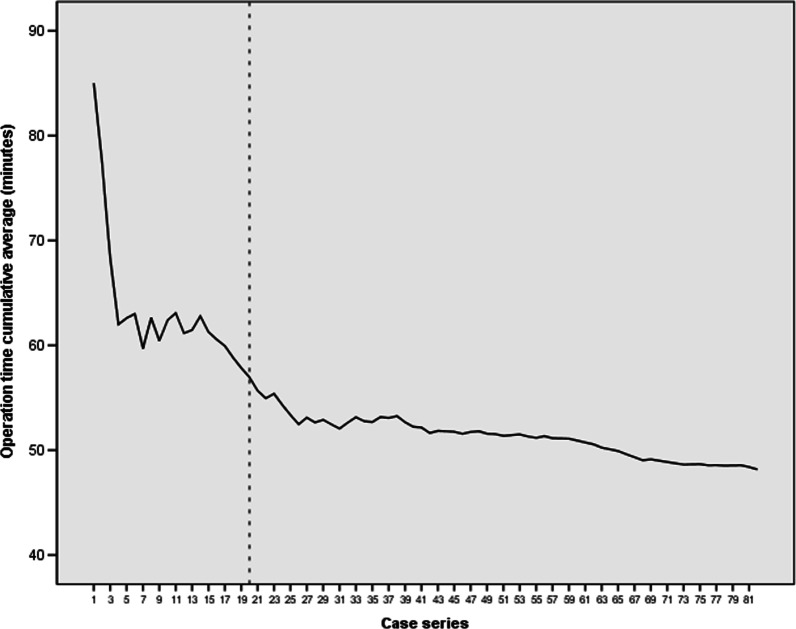
Linear and log regression analyses. Operation time = 58.825—(0.181 × [case number]) (p < 0.001)

According to the cumulative study, the cumulative average operation time appeared to plateau after 20 cases. The mean operation time were most significantly different between the earlier 20 cases and the later 62 cases (56.95 [95%CI, 49.12–64.78] minutes in the early group versus 45.34 [95% CI, 42.45–48.22] minutes in the late group; p = 0.008, non-parametric Mann–Whitney U test) (Fig. 5).

**Fig. 6:**
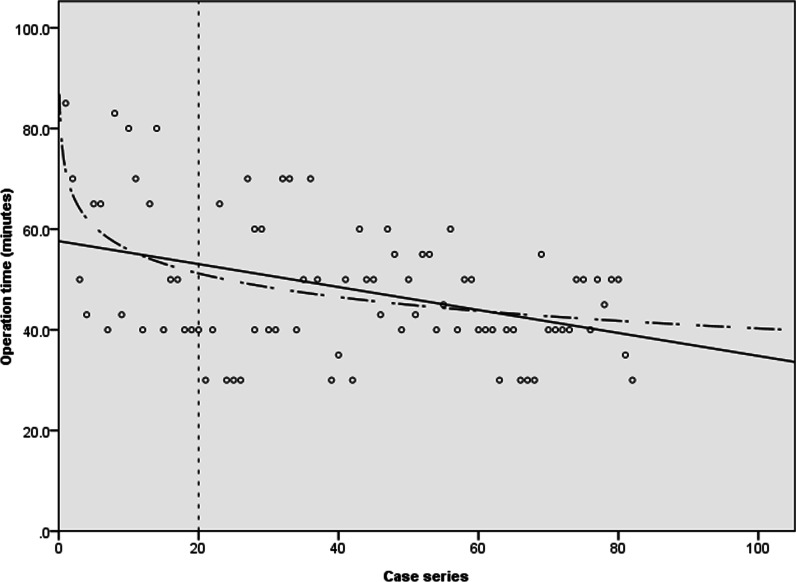
Cumulative study of the average operation time

According to linear regression analyses, the operation time was obtained by the formula: operation time = 58.825—(0.181 × [case number]) (p < 0.001), suggesting that the operation time was significantly reduced with the accumulation of operations performed. Furthermore, the proportional constant, − 0.181, means that the operation time decreased straightly even in cases where surgeons had limited surgical experience (Fig. 6).

### Baseline characteristics between the early and late groups

Demographic data and clinical baseline characteristics were not different between the two groups (Table 1).

**Table 1:** Demographic data and clinical baseline characteristics between the two groups

	Early group (n = 20)	Late group (n = 62)	OR or difference	95% CI	P value
Age	41.15 ± 15.44	40.43 ± 15.43	− 0.72 ± 4.82	− 9.030–10.480	0.882^†^
Male ratio	14 (70.0%)	38 (61.3%)	1.750	0.482–6.351	0.520^‡^
Smoking status	7 (35.0%)	19 (30.6%)	0.743	0.199–2.779	0.658^‡^
Alcohol consumption (g/week)	0.42 (95% CI 0.08–0.76)	0.43 (95% CI 0.097–0.761)	0.01 ± 0.23	− 0.469–0.452	0.820^§^
Height (cm)	170.67 ± 11.37	168.32 ± 9.17	2.35 ± 3.22	− 4.163–8.885	0.470^†^
Weight (kg)	68.92 ± 15.86	70.04 ± 11.81	1.12 ± 4.35	− 9.926–7.680	0.798^†^
Body mass index (kg/m^2^)	23.46 ± 3.72	24.79 ± 4.22	1.33 ± 1.25	− 3.847–1.191	0.293^†^
Diabetes	1 (5.0%)	5 (8.1%)	2.000	0.617–23.960	0.578^‡^
Hypertension	3 (15.0%)	15 (24.2%)	2.267	0.481–10.680	0.294^‡^
Previous block	9 (45.0%)	39 (62.9%)	3.056	0.838–11.136	0.086^‡^
Trauma history	3 (15.0%)	9 (14.5%)	0.944	0.167–5.339	0.948^‡^
Symptom duration (weeks)	3.87 (95% CI 1.84–5.90)	1.63 (95% CI 0.67–2.60)	2.24 ± 1.06	0.949–4.378	0.092^§^
Dominant symptom, back pain/leg pain	8/12	18/44	2.133	0.556–8.187	0.265^‡^

*CI* confidence interval, *OR* odds ratio

^†^Independent t-test, ‡Pearson’s Chi square test, §Mann–Whitney U test

Baseline characteristics determined by preoperative MRI and intraoperative findings, also, were not differ significantly between the two groups (Table 2).

**Table 2:** Baseline characteristics determined by preoperative magnetic resonance imaging and intraoperative findings

	Early group (n = 20)	Late group (n = 62)	OR or difference	95% CI	P value
Surgical level, L3-4/L4-5/L5-S1	2/8/10	4/14/44			0.111^†^
Pfirmann grade, I/II/III/IV	0/4/12/3	0/18/38/7			0.763^†^
High intensity zone	6 (30.0%)	22 (35.5%)	1.436	0.391–5.269	0.585^‡^
Morphology of disc, bulging/protruded/extruded	2/11/9	8/35/17			0.130^†^
Location of herniation, central/right/left	6/7/7	20/13/29			0.092^†^
Degree of canal compromise, mild/moderate/severe	13/7/0	43/15/0			0.208^†^
Degree of nerve compression, abutting/displace/near obliteration/obliteration	8/7/4/1	34/21/6/1			0.264^†^
Herniated disc volume (mm^3^)	0.31 ± 0.11	0.30 ± 0.14	0.01 ± 0.04	− 0.08–0.09	0.898^‡^
Degree of stenosis, none/mild/moderate/severe	12/7/1/0	42/19/1/0			0.450^†^
Intraoperative adhesion, mild/moderate/severe	2/2/16	3/20/29			0.249^†^

*CI* confidence interval, *OR* odds ratio

^†^Pearson’s Chi square test, ‡independent t-test

### Surgical outcome between the early and late groups

The overall surgical complication rate was 8.5% and surgical failure or recurrence rate was 17.1% during 6 months after surgery. Although the operation time was significantly shorter in the late group, the surgical outcomes, including hospital stay, return to work, surgical complication rate, and surgical failure or recurrence rate, were not different. In particular, surgical complication rate (10.0% in the early group versus 8.1% in the late group) and surgical failure or recurrence rate (15.0% in the early group versus 17.7% in the late group) were similar between the two groups (Table 3). Surgical complications in the early group included 1 patient with transient nuchal pain during the procedure and 1 patient with transient motor weakness; whereas, surgical complications in the late group included 3 patients with transient nuchal pain during the procedure, 1 patient with transient motor weakness, and 1 patient with dura puncture during the procedure.

**Table 3:** Surgical outcomes between the two groups

	Early group (n = 20)	Late group (n = 62)	OR or difference	95% CI	P value
Operation time (min)	56.95 (95% CI, 49.12–64.78)	45.34 (95% CI, 42.45–48.22)	11.61 ± 4.01	3.353–19.872	0.008^†^
Hospital stay (days)	3.7 ± 0.9	3.5 ± 1.2	0.2 ± 0.8	− 0.749–1.354	0.764^‡^
Return-to-work (days)	15.5 ± 7.0	15.0 ± 5.2	0.5 ± 2.0	− 4.429–5.783	0.848^‡^
Complication	2 (10.0%)	5 (8.1%)	0.950	0.055–16.293	0.744^§^
Failure or recurrence	3 (15.0%)	11 (17.7%)	0.083	0.009–0.781	0.904^§^
Additional block	2 (10.0%)	6 (9.7%)	0.947	0.120–7.457	0.678^§^
Revision surgery	1 (5.0%)	5 (8.1%)	2.000	0.167–23.960	0.578^§^

*CI* confidence interval, *OR* odds ratio

^†^Mann–Whitney U test, ‡independent t-test, §Pearson’s Chi square test

### Clinical outcome and radiological outcome between the early and late groups

The overall patient satisfaction rate was 58.5% according to Odom’s criteria at final follow-up. Clinical outcomes, including VAS for low back pain or leg pain and patient satisfaction according to Odom’s criteria, were not significantly different between the two groups (Table 4).

**Table 4:** Clinical outcomes between the two groups

	Early group (n = 20)	Late group (n = 62)	OR or difference	95% CI	P value
*VAS for low back pain*					
Preoperative	5.60 ± 1.82	5.29 ± 1.68	0.31 ± 0.546	− 0.791–1.422	0.568^†^
1 week	3.45 ± 1.79	3.00 ± 1.00	0.45 ± 0.45	− 0.462–1.366	0.324^†^
1 month	2.71 ± 1.40	2.47 ± 1.74	0.24 ± 0.54	− 0.878–1.342	0.667^†^
6 months	2.89 ± 1.69	2.80 ± 1.32	0.09 ± 0.62	− 1.194–1.371	0.887^†^
*VAS for leg pain*					
Preoperative	6.15 ± 1.63	6.05 ± 1.75	0.10 ± 0.53	− 0.977–1.174	0.847^†^
1 week	4.00 ± 1.56	3.81 ± 2.09	0.019 ± 0.58	− 0.983–1.367	0.743^†^
1 month	3.53 ± 2.15	3.18 ± 2.60	0.35 ± 0.82	− 1.320–2.022	0.670^†^
6 months	3.56 ± 1.67	3.60 ± 2.35	0.04 ± 0.90	− 1.917–1.821	0.961^†^
*Odom’s criteria*					
1 week, Excellent/good/fair/poor	2/11/6/1	8/29/24/1			0.577^‡^
1 month, Excellent/good/fair/poor	3/8/9/0	17/20/25/0			0.383^‡^
6 months, Excellent/good/fair/poor	2/9/7/2	14/23/23/2			0.231^‡^

*CI* confidence interval, *OR* odds ratio, *VAS* visual analog scale

^†^Independent t-test, ‡Pearson’s Chi square test

Also, radiological outcomes, including disc height, neutral segmental angle of surgery level, range of motion of surgery level, and total lumbar lordosis, were not significantly different between the two groups at 6 months after surgery (Table 5).

**Table 5:** Radiological outcomes between the two groups (independent t-test)

	**Early group** **(n = 20)**	**Late group** **(n = 62)**	**OR or difference**	**95% CI**	**P value**
*Disc height (mm)*					
Preoperative	17.31 ± 3.74	18.49 ± 1.85	1.18 ± 0.89	− 2.03–1.93	0.466^†^
6 months	17.24 ± 1.65	18.25 ± 1.42	1.01 ± 1.55	− 2.34–1.91	0.260^†^
*Segmental angle (°)*					
Preoperative	7.90 ± 5.81	8.82 ± 4.54	0.92 ± 1.67	− 1.32–2.48	0.586^†^
6 months	7.24 ± 4.21	8.32 ± 4.15	1.08 ± 1.826	− 4.88–2.72	0.560^†^
*Range of motion (°)*					
Preoperative	4.86 ± 3.99	6.64 ± 4.01	1.78 ± 1.41	− 4.63–1.07	0.214^†^
6 months	3.19 ± 2.72	5.82 ± 4.75	2.63 ± 2.093	− 6.90–1.63	0.217^†^
*Total lumbar lordosis (°)*					
Preoperative	33.31 ± 15.95	34.92 ± 16.05	1.61 ± 5.22	− 12.21–8.98	0.759^†^
6 months	34.74 ± 7.35	36.47 ± 12.40	1.73 ± 4.81	− 11.73–8.26	0.722^†^

CI: Confidence interval, OR: Odds ratio

^†^ independent t-test

## Discussion

The concept of SELD is an intermediate step between intervention (such as, nerve block or neuroplasty) and surgery (such as, microdiscectomy or full endoscopic surgery). In other words, when nerve block is ineffective or a more effective procedure is needed, but the operation is over-treatment, the SELD can be an option for disc herniation. In terms of this position of SELD, its indication or role is similar with annular modulation or annuloplasty. However, the advantages of SELD includes availability of real endoscopic vision of target lesion, direct ablation of herniated disc, adhesiolysis, and drug injection. In addition, in terms of simple minimally invasive procedure under local anesthesia, the indications of SELD need not to be too heavy like a microdiscectomy or full endoscopic surgery.

According to previous studies, the clinical or surgical outcomes of SELD were found to be favorable in various lumbar spine diseases [3–7, 12–17]. In particular, several recent papers regarding the clinical outcome of SELD for disc herniation have reported that the clinical outcome was favorable as there was significant improvement in low back pain or radiating leg pain, patient satisfaction rate of more than 70%, and low rates of surgical failure or recurrence [8, 18–21].

However, according to the result of this study and author’s previous report, the clinical outcome was inconsistent with that in previous reports as the patient satisfaction rate was 58.5% according to Odom’s criteria and the surgical failure or recurrence rate was 17.1% during 6 months of follow-up [9]. This result was not favorable compared to not only previous studies on SELD but also the results of other surgical techniques for lumbar disc herniation [22–24].

After considering the reason for these discordances, we hypothesized that surgeon’s learning curve of SELD could affect the outcome. In other words, we speculated that the result may not be favorable in the early stage of clinical application compared to that at the adapted stage; as a result, the overall clinical outcome could be unfavorable.

SELD is considerably different from conventional microsurgery or full endoscopic surgery because of the different access route and equipment used. The obstacles in starting SELD include different access methods via the sacral hiatus, unfamiliarity to a steerable guide catheter, difficulty in reaching the target lesion, the use of a very narrow and magnified endoscopic view, the presence of a vague or obscured view owing to epidural bleeding or fat, fear of intradural insertion of a catheter, or uncertainty of successful decompression. The trainee should have worked on at least a certain number of cases to become accustomed to the trans-sacral approach and very narrow two-dimensional steerable endoscopic vision. These barriers may pose challenges for a surgeon at the beginner stage and might result in a steep learning curve and cause unfavorable and inconsistent clinical outcomes.

Operation time is a major parameter to assess the technical proficiency of surgeons [25]. A trend of operation time is an effective statistical tool to assess whether a trainee has achieved acceptable proficiency [25]. Surgeon’s comfort and technical proficiency is correlated to a decrease in procedure length in chronological case series, and the traditional evaluation of the learning curve has focused on operation time according to the number of cases [26].

In our study, as the number of cases accumulated, the operation time was shortened as a result of familiarity with the surgical technique. The cumulative analysis identified a threshold of 20 cases after which the operation time was consistent. In other words, the operation time approached an asymptote in the 20th case and decreased from a mean 56.95 min in the initial 20 cases to a mean 45.34 min in the later 62 cases (decrease of 20%). On the basis of the asymptote point, we found out that the learning curve of SELD is similar to 10–30 cases of other spinal surgeries, such as microsurgery using tubular retractor or full endoscopic surgery [27–31]. Also, based on the 20% decrease of operation time and proportional constant of -0.181 in the formula of operation time, the rate of decline is not steep compared to the 23% to 58% decrease in operation time during the initial series of cases between the 10th and 30th case of other minimally invasive spinal surgeries [27–31]. These findings imply that the entry barriers for beginners to start SELD are easier or similar compared to those for other techniques.

Another clinically relevant parameter used to assess proficiency of surgeon through the learning curve is the complication or failure rate. Incompetence is inevitable when learning a new surgical technique, particularly minimally invasive surgery; thus, majority of surgery-related complications, failure, or conversions to open techniques usually occurred within the beginner stages of the learning process [26]. The lack of clear anatomic knowledge or orientation and unfamiliarity of new instruments appears to be a significant limitation, and this may cause serious injury to neurologic structures or unintended adverse events in the initial series of patients [32]. Multiple studies on minimally invasive spine surgery have reported that the complication rate is higher and the clinical outcome is poorer at the beginner level than at the expert level [23, 27, 33, 34].

However, in our study, both the clinical outcomes and the surgical outcomes, including complication rate and failure or recurrence rate, were similar between the early and late groups. Based on previous studies, the complication rate of 10% in the early group was favorable compared to the complication rate of 14–40% in the novice stage of other minimally invasive spinal surgeries [26, 35–37]. Furthermore, the overall incidence of complications was only 8.5% (7 of 82 patients) and complications were only mild-to-moderate. Among the complications, the transient nuchal pain is related to increase of intracranial pressure due to excessive epidural injection of normal saline for irrigation during surgery. In addition, we speculate that transient motor weakness is related to temporary irritation of nerve root by catheter or laser ablation. These findings suggest that, compared to other minimally invasive spinal surgeries, SELD is relatively easy to learn and is a safe procedure with less complications.

This study has several limitations. Because of its retrospective study design, it was impossible to control for all variations. Moreover, the number of patients in the final cohort was relatively small, and the research was conducted at a single center. However, this single-center study could maintain the quality of follow-up and exclude the factor related to the diversity of surgeons.

To the best of our knowledge, this study is the first to evaluate the learning curve and related outcome of SELD in lumbar disc herniation. More complete studies with a prospective design are required to establish SELD as an easy to learn and safe procedure.

## Conclusion

Based on the operation time and outcomes, the learning curve of SELD was not as difficult as that of other minimally invasive spinal surgeries. According to this result, we believe that SELD is an easy technique to start with safety for novice surgeons. However, further studies are necessary to elucidate the influencing factors on the clinical outcomes of SELD.

## Data Availability

The datasets used and analyzed during this study are available from the corresponding author upon reasonable request.

## Abbreviations

CI: Confidence interval
MRI: Magnetic resonance imaging
OR: Odds ratio
SELD: Trans-sacral epiduroscopic laser decompression
VAS: Visual analogue scale
